# Supplementary material for: Expression and Characterization of a Cold-Adapted Alginate Lyase with Exo/Endo-Type Activity from a Novel Marine Bacterium Alteromonas portus HB161718T
Source: Mar Drugs. 2021 Mar 17;19(3):155. doi: 10.3390/md19030155 (PMC8002439; doi:10.3390/md19030155)
Supplement: Supplementary file 1 [file marinedrugs-19-00155-s001.pdf]

marine drugs

Article

Expression and Characterization of a Cold-adapted Alginate Lyase with Exo/endo-type Activity from a Novel Marine Bacterium *Alteromonas portus* HB161718^{T}

Huiqin Huang [1,4*], Shuang Li [1,3], Shixiang Bao [1,4], Kunlian Mo [1,4], Dongmei Sun [3] and Yonghua Hu [1,2,4,*]

1 Institute of Tropical Bioscience and Biotechnology, Hainan Institute for Tropical Agricultural Resources, CATAS, Haikou 571101, China; 1468664229@qq.com (S.L.); bsxitbb@163.com (S.B.); mokunlian@163.com (K.M.)
2 Laboratory for Marine Biology and Biotechnology, Pilot National Laboratory for Marine Science and Technology, Qingdao 266071, China
3 College of Life Science and Technology, Heilongjiang Bayi Agricultural University, Daqing 163000, China; sdmlzw@126.com (D.S.)
4 Hainan Provincial Key Laboratory for Functional Components Research and Utilization of Marine Bioresources, Haikou 571101, China
* Correspondence: huanghq21@163.com (H.H); huyonghua@itbb.org.cn (Y.H.);
Tel.: +86-898-66890695 (H.H.); +86-898-66890671 (Y.H.)

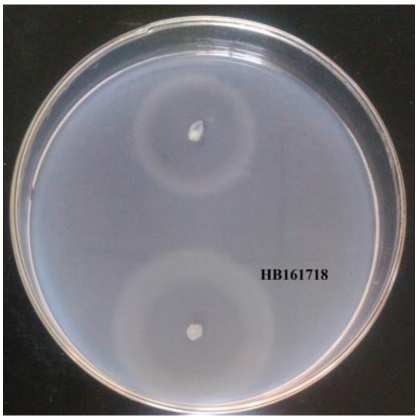

Figure S1. Gellation reactions observed on the plate covered by $CaCl_2$ solution

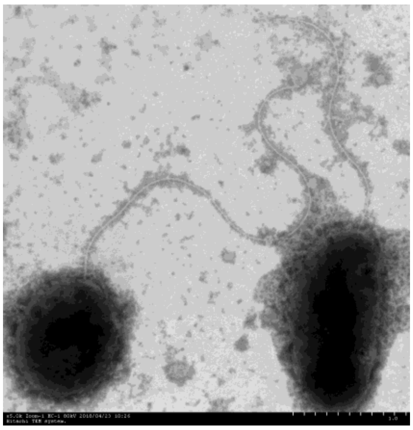

Figure S2. Transmission electron micrograph of cells from a 12-hour-old culture on marine agar 2216 of strain HB161718^{T}. Bar, 1 μm.

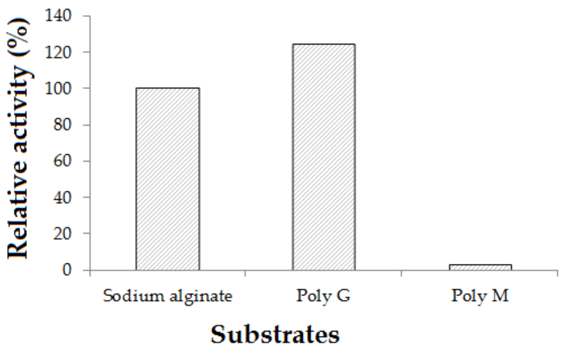

Figure S3. Substrate specificity of the recombinant enzyme ALg2951
